# Daily supplementation with GrandFusion^®^ improves memory and learning in aged rats

**DOI:** 10.18632/aging.101209

**Published:** 2017-03-24

**Authors:** Jin Yu, Hong Zhu, Stephen Perry, Saeid Taheri, Mark S. Kindy

**Affiliations:** ^1^ Department of Pharmaceutical Sciences, College of Pharmacy, University of South Florida, Tampa, FL, USA; ^2^ NutriFusion®, LLC, Atlanta, GA, USA; ^3^ James A. Haley VA Medical Center, Tampa, FL, USA; ^4^ Shriners Hospital for Children, Tampa, FL, USA

**Keywords:** aging, inflammation, oxidative stress, diet, memory

## Abstract

Studies have shown that supplementation with extracts from various sources, including fruits and vegetables reverse the age-related changes in movement and cognition. We hypothesized that these beneficial effects result from the presence of anti-oxidants and anti-inflammatory compounds in the fruits and vegetables that contribute to reduced oxidative stress, inflammation and cell death while potentially enhancing neurogenesis. The present study was performed to determine the impact of supplementation with GrandFusion^®^(GF) to aged Fisher 344 rats for 4 months to determine the impact on attenuation or reversal of the age-related deficits. When the aged rats consumed a diet enriched with the extracts the results showed an improved motor performance, and enhanced cognitive functions. In addition, the rats showed reduced oxidative stress and inflammation, and enhanced neurogenesis, Nrf2 and anti-oxidant expression. The effect of GF extracts on the augmentation of memory and learning is significant and may function through the modulation of antioxidant enzymes, signaling pathways and additional mechanisms to improve the aging process. These studies further support the recommendation of USDA for the consumption of fruits and vegetables to improve healthy aging.

## INTRODUCTION

Aging is a multifaceted process that results in increases in inflammation, oxidative stress (OS) and contributes to cellular senescence, eventually leading to a decline in organ function and death [1-4]. Aging and other neurological or neurodegenerative disorders result in deficits in cognitive and motor performance as a result of enhanced susceptibility to the long-term effects of increased OS and inflammation [5,6]. A decrease in endogenous antioxidant mechanisms with aging increases the vulnerability of the brain to oxidative damage as a result of exacerbated OS [7]. In addition, the aged brain is much more susceptible to the impact of inflammation compared to the young brain [8]. Aging results in an increase in inflammatory mediators (i.e., cytokines, chemokines, etc.) liberated by microglia and astrocytes in the brain and an infiltration of peripheral inflammatory cells that contribute to the cognitive and physical deficits [9]. These changes in OS and inflammation result in alteration in membrane structure, modifications in DNA, RNA, protein and lipids that contribute to age-related adjustments in brain vulnerability [10,11].

Because of the potential impact of OS and inflammatory vulnerability in aging, research has focused on approaches to ameliorate these effects and attenuate the deficits in cognitive function [12-14]. A number of studies have concentrated on the use of exogenous and synthetic anti-oxidants and anti-inflammatory agents, including plant-derived compounds that protect against stress-related phenomenon [15,16]. Over the years, a number of compounds have been isolated from various plant species that protect the plants from OS and inflammation [17,18]. Studies have identified polyphenols, carotenoids, flavonoids, and sulfur compounds as natural anti-oxidants and anti-inflammatory agents that protect the plant from susceptibility to harmful conditions [19]. Finally, studies have been investigating the role of these compounds in reducing the age-related sensitivity of the brain to OS and inflammation [20].

Our previous studies have shown that dietary supplementation with GrandFusion® (GF), which is a nutritional supplement that contains the natural ingredients from whole fruits and vegetables demonstrated anti-oxidant, anti-inflammatory, and neuroprotective properties [21]. We showed that when mice were fed a diet supplemented GF for two months followed by cerebral ischemia were protected from infarct damage in the brain and demonstrated improved behavioral outcomes. In addition, the diet attenuated inflammatory processes by reducing microglial and astrocytic activation. The study implicated GF in the neuroprotective effects against the impact of cerebral ischemia and reperfusion injury.

Previous studies have shown that extracts from blueberries or strawberries can attenuate age-related motor and cognitive deficits in aged rat [22]. When young rats were fed a diet enriched in AIN-93 supplemented with spinach or strawberry extracts, the animals did not show age-related changes in cognitive function when compared to control rats [23]. In addition, older rats fed diets enriched in blueberry or strawberry extracts over an 8 week period, resulted in a reversal of cognitive and behavioral deficits [24]. Other studies using NT-020 (enriched in polyphenols from blueberry extracts) reduced inflammation, OS and improved Nrf-2 signaling [25]. These studies suggest that extracts from fruits and vegetables contain compounds that not only reduce age-related inflammation and OS, but help to enhance neurogenesis in the brain that may contribute to improved outcomes [25].

The present study was carried out to further assess the behavioral and biochemical benefits of a diet rich in vegetables and fruits. Our results demonstrate that these diets show a positive influence on behavioral outcomes associated with the aging phenomenon [21]. In addition, advantageous changes in biochemical parameters such as inflammation, reactive oxygen species (ROS) and cellular signaling are present in animals fed a diet enriched in fruits and vegetables. Finally, we have demonstrated that the supplemented diets enhanced neurogenesis in the brain of aged animals. These data suggest that these diets can affect the pronounced changes seen in aging to slow the process and improve outcome.

## RESULTS

### Overall appearance and weights of animals in the study

**Figure 1:**
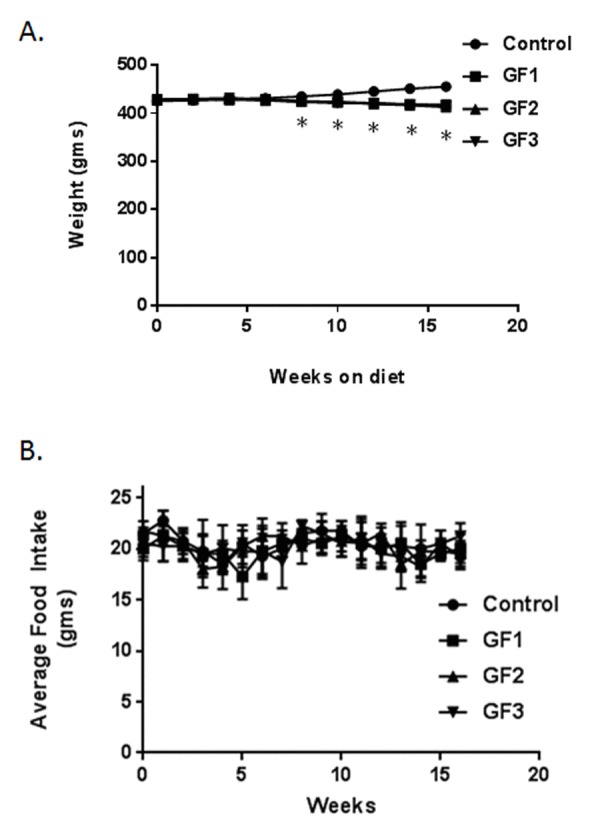
Animal weights and food intake Animals were fed a standard diet of NIH-31 or diets enriched in GrandFusion (NF-216,GF1; NF-316, GF2; or NF-416, GF3). **(A)** Animals were weighed starting on month 18 and for 16 weeks after starting the diet. **(B)** Average food intake was determined every week after start of the diets. Values are means, with their standard errors represented by vertical bars. N = 20 per group. *P<0.001.

Mean body weights of all groups over the 16 weeks of experimental period were illustrated in Figure 1A. The control group showed a steady weight over the time course of the study. The rats on the GrandFusion® (GF) diets, showed a slight decrease in weight with the diets that was significant. The calorie content between the different diets was the same and this was not the result of the body changes. All four groups are present, there is significant overlap and appear together. The average food intake was also determined in the rats over the course of the 16 weeks (Figure 1B). In that time, there were no significant differences in food consumption between the control rats and the rats on the GF diets.

### Improvement in psychomotor test in aged rats

**Figure 2:**
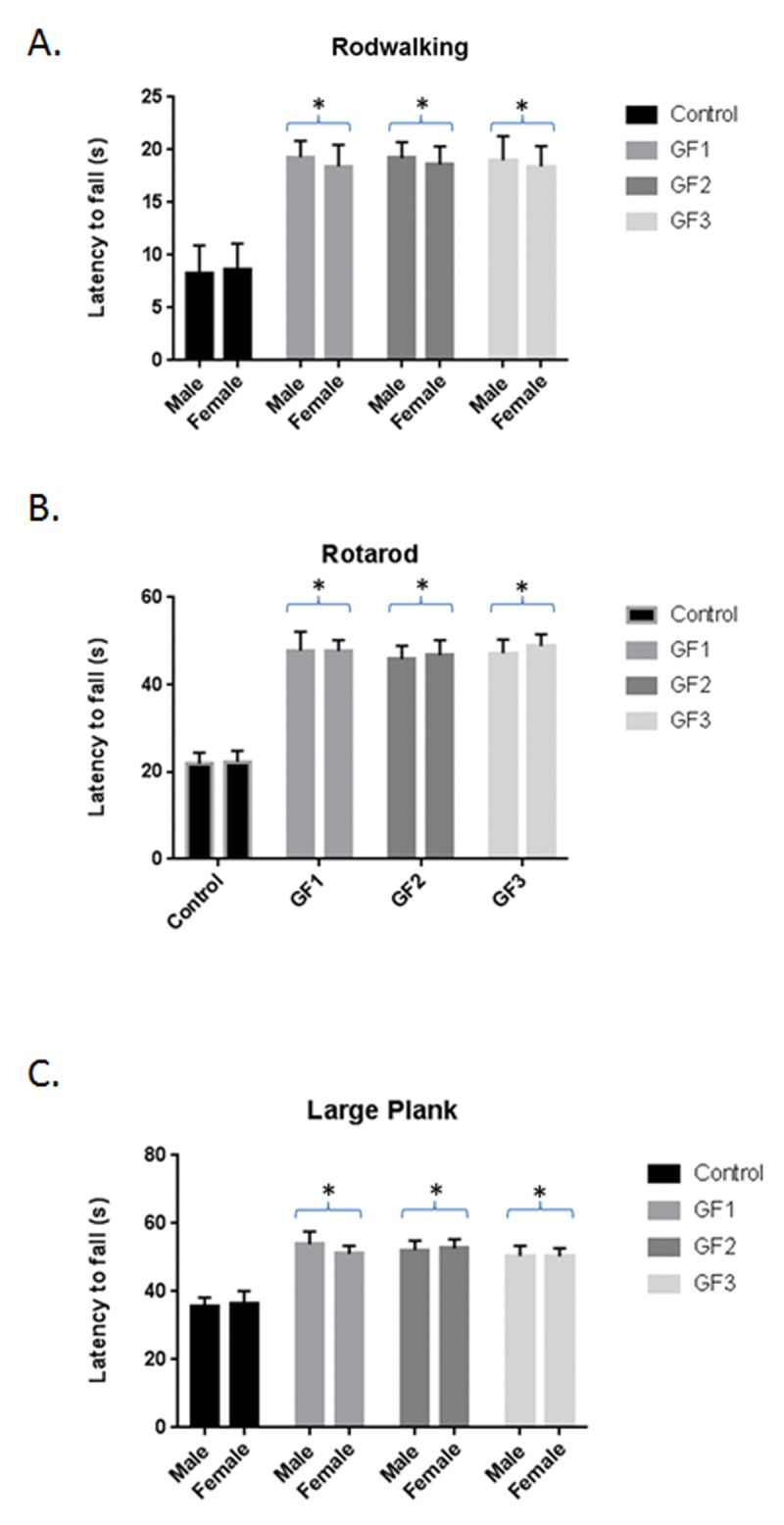
Behavioral testing following diet feeding Performance (latency to fall, in seconds) on rod walk **(A)**, rotarod **(B)** and large plank **(C)** for the various diet groups. Male and female 18 month old Fisher 344 rats were fed chow with and without GF diets and examined for various behavioral tests. Values are means, with their standard errors represented by vertical bars. N = 10 per group. *P<0.001.

The aged rats that consumed the GF diets performed significantly better on the behavioral tests than did control aged rats; and all of the GF diets resulted in similar effectiveness for all the behavioral endpoints. In addition, both male and female aged rat groups showed improved performance on tests of psychomotor performance compared with control aged rats (regular diet). On the rod walk (Fig. 2A), latency to fall was significantly higher in the GF groups than in the control group (*P* < 0.05) the aged males showed a slightly better improvement in the rod walk compared to the females but this was not significantly different. In the rotarod test (Fig. 2B) latency to fall was significantly better in the GF groups than in the control group (*P* ≤ 0.05). There was no difference between the aged male and aged female rats. Finally, group differences were seen on the large plank test (Fig. 2C). The GF groups showed significant improvement when compared with the control group on the large plank assay. Therefore, GF supplementation improved performance on motor tests, which rely on balance, coordination, and strength.

### Enhanced cognitive testing in aged rats

**Figure 3:**
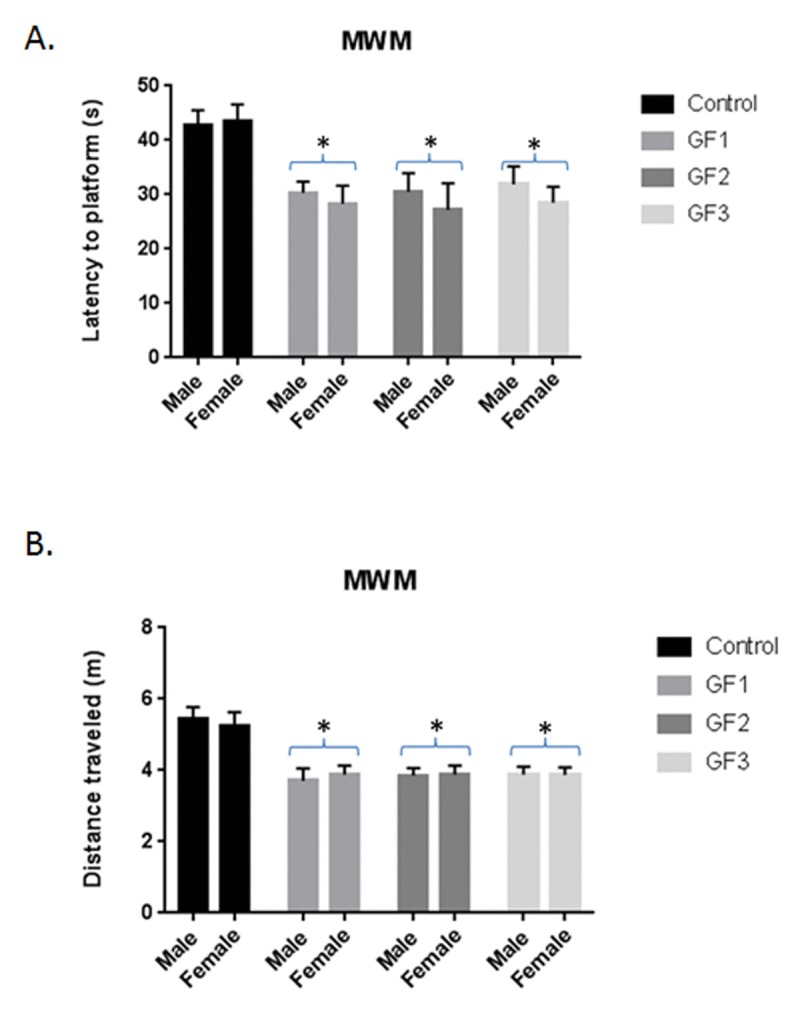
Morris water maze testing Morris water maze performance assessed as latency in seconds **(A)** and distance **(B)** to find the hidden platform over days 4 and 5 of testing for animals in control, GF1, GF2 and GF3 diet groups. After 16 weeks on the GF diets, the animals were subjected to Morris water maze. The final trial day is presented as seconds to find the hidden platform. Both males and females were used in the studies. Values are means, with their standard errors represented by vertical bars. N = 10 per group. *P<0.001.

When examining cognitive performance, the GF groups showed improved performance over the control group in the Morris water maze test (Fig. 3A and B). We performed *t*-tests between the trial latencies and distances for each group to determine whether the different groups significantly improved their performance, which would demonstrate improved working memory. The GF groups showed significant (*P* < 0.05) differences in latencies (Fig. 3A) and distances (Fig. 3B) compared to the control group. These differences were not due to learning ability or swim speed because there were no differences between groups on these parameter (data not shown). There were no differences between the male and female aged rats in the GF groups and subsequent data analysis combined the two groups.

### Enhanced neurogenesis

**Figure 4:**
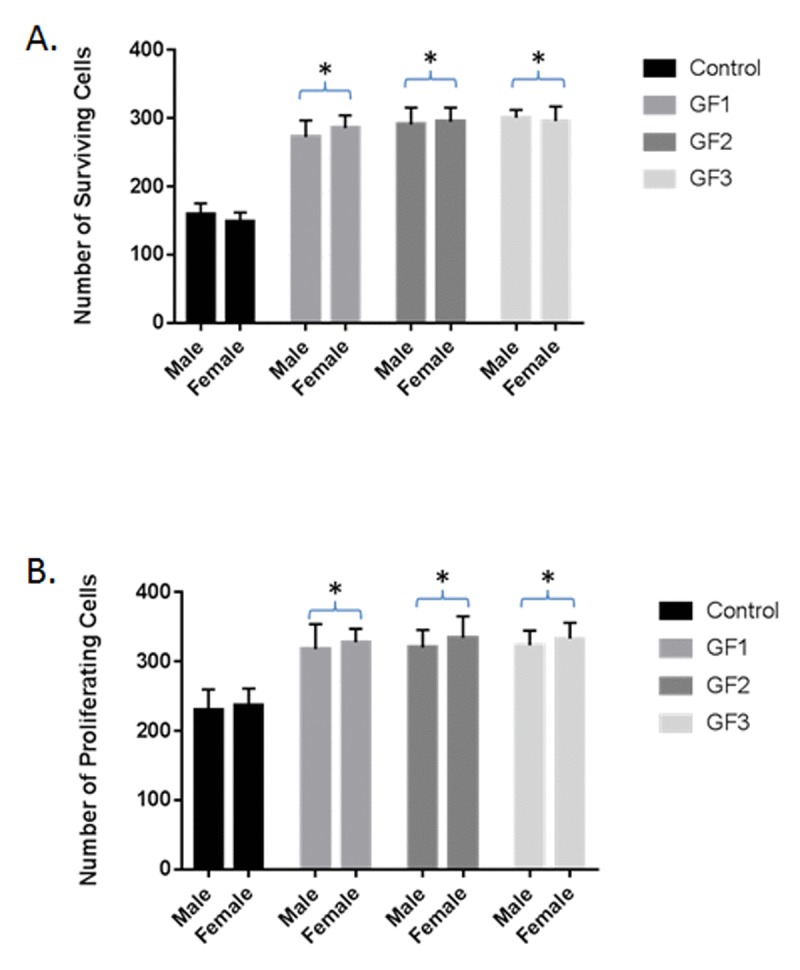
Neuroprotection and neurogenesis The number of surviving **(A)** and proliferating **(B)** precursor cells in the dentate gyrus of the hippocampus of rats in the control, GF1, GF2 or GF3 diet groups. Values are means, with their standard errors represented by vertical bars. N = 20 per group. *P<0.05.

Differences were seen in cell survival among the diet groups compared to the control group (*P* < 0.05; Fig. 4A), with rats in the GF groups showing a significant increase in the number of cells surviving in the dentate gyrus of the hippocampus compared with the control diet group (*P* < 0.05). In addition, the rats in the GF groups also showed higher numbers of proliferating precursor cells (Fig. 4B), these differences did reach significance (*P* < 0.05). In both measures, rats in both the male and female GF diet groups were significantly different from the control group (Fig. 4A and B). Correlations between neurogenesis and cognitive performance revealed that, as the number of proliferating cells increased, the mean difference in latency was shorter in the GF groups (Fig. 3 and 4). The difference in the score is a measure of working memory, and the score implies that the rats were able to find the platform more quickly, remembering where it was on earlier trials. All of the GF groups showed significant correlations between proliferation and working memory performance (*P* < 0.05). In addition, the cognitive performance did significantly correlate with surviving cells, and all of the motor tests showed a positive correlation with neurogenesis.

### Reduced oxidative stress in diet treated rats

**Figure 5:**
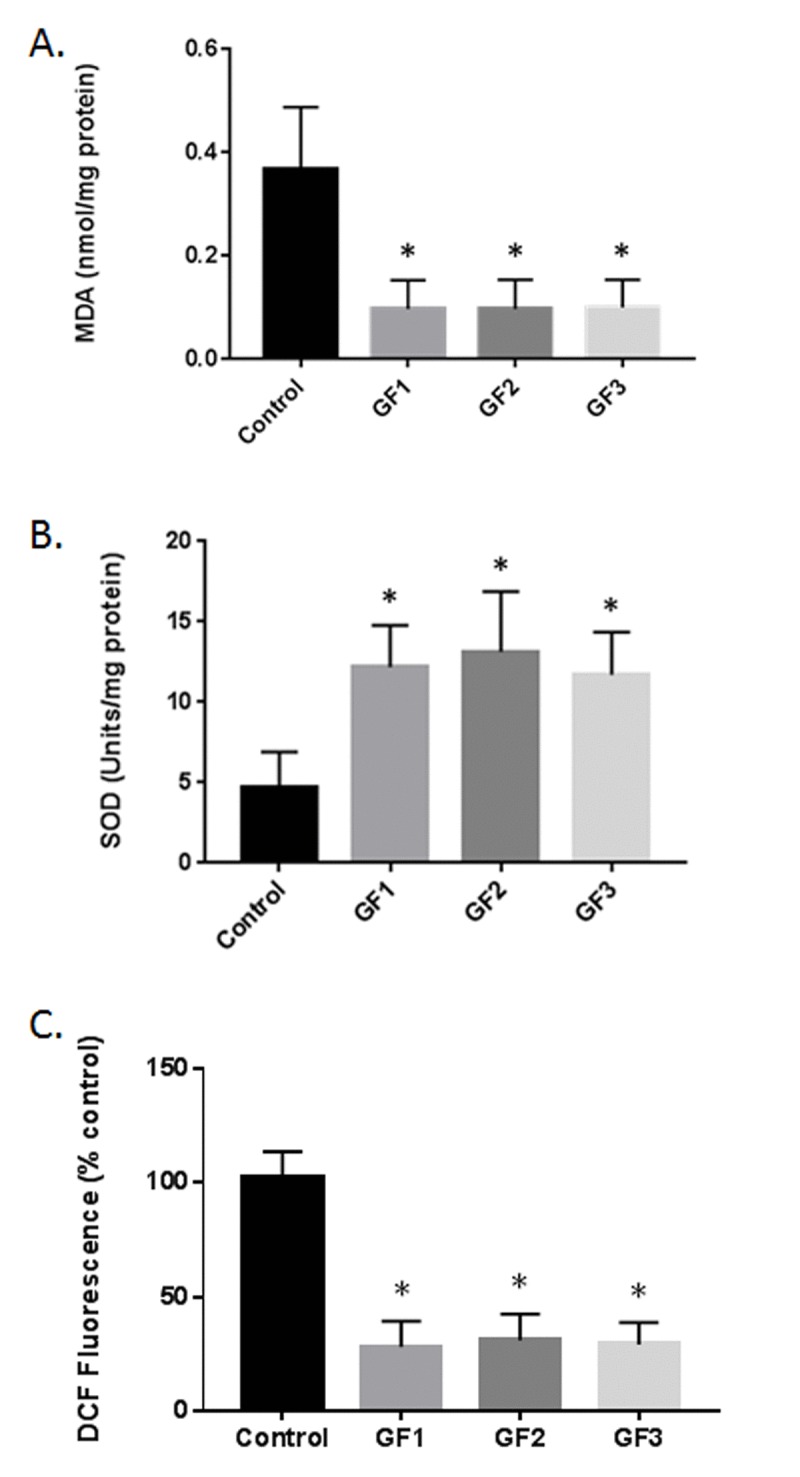
Reduction in oxidative stress following GF diets **(A)** MDA levels in the brains of aged rats subjected to control or GF diets. **(B)** SOD activity in the brains of aged rats following feeding control diets or diets enriched with GF formulas. **(C)** DCF assay in the brains of aged rats following feeding control diets or diets enriched with GF formulas. N = 20 per group. P<0.001.

Supplementation of the GF diets demonstrated changes in malondialdehyde (MDA) or thiobarbituric acid reactive substances (TBARS) (Fig. 5A). The diets showed a significant effect on MDA/TBARS levels in the brain in control aged and GF supplemented animals. Significant differences were present in the brain with all of the diets demonstrating an effect in reducing lipid peroxidation products. The results showed that the control rats had greater values when compared with the GF groups. The diets showed ∼75% reduction in TBARS. DCF measurements in the brain of aged animals treated with GF diets lead to a reduction in free radical levels compared with control aged animals (Fig. 5C). The GF diets showed improvement with approximately ~70% reduction in free radicals. The impact of the diets on superoxide dismutase (SOD) activity was detected in different groups. Aged rats demonstrated a significant decrease in SOD activity in the brain at the end of the study (Fig. 5B). In the GF supplemented aged rats, there was an increase in the SOD activity compared with the control rats. The diets caused an increase in SOD activity at the end of the experiment. These results suggest that aging exacerbates oxidative stress in the brain and the diets where capable of suppressing the oxidative stress and enhancing anti-oxidant activity.

### Reduced inflammatory markers in diet fed aged rats

**Figure 6:**
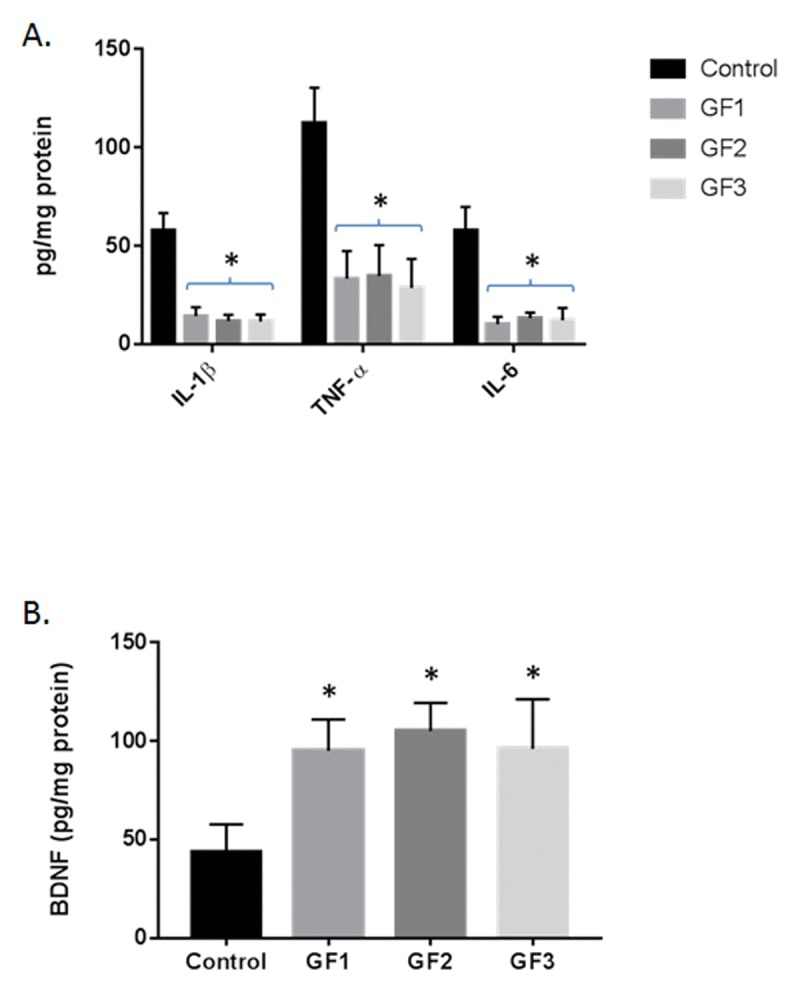
Cytokine and BDNF levels in the brains of aged rats **(A)** IL-1β, TNF-ɑ and IL-6 were measured in the brains of aged rats subjected to control or GF diets. **(B)** BDNF was measured in the brains of aged rats subjected to control or GF diets. N = 20 per group. P<0.001.

Quantification of inflammatory markers (IL-1β, TNF-ɑ and IL-6) by ELISA in the brain showed that aged animals fed the GF diets exhibited significantly lower overall cytokine activity than the control aged rats (Fig. 6A, *P* < 0.01). Consumption of a GF diets in the aged rats was associated with decreased expression of IL-1β, TNF-ɑ and IL-6. Interestingly, all of the GF diet supplementation showed a significant attenuated of cytokine expression. Additionally, the brains were analyzed for the expression of brain derived neurotrophic factor (BDNF) and the GF diets showed a significant increase in BDNF levels compared to the control diet rats (Fig. 6B). Overall, there is a significant decrease in inflammatory markers with an increase in trophic factor expression.

### Microglial and astrocyte activation following GF diets

**Figure 7:**
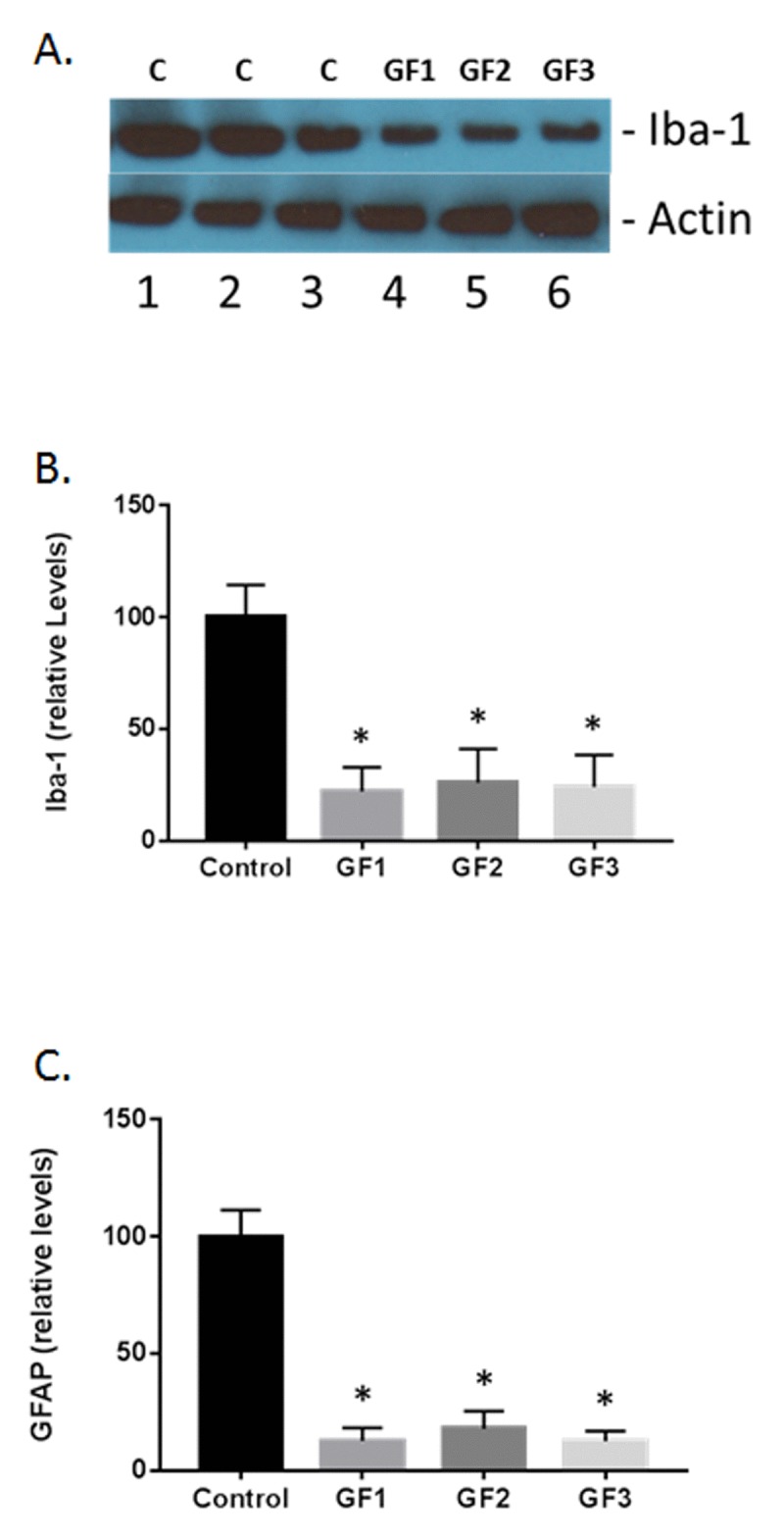
Measurement of glial activation in the aged brain and brains of animals fed GF diets **(A)** Representative western blot of iba-1 levels in the brains of aged rats subjected to control or GF diets. C, control; GF1, GF2, GF3, GF diets. Actin is used as a control for loading. **(B)** Quantified levels of iba-1 in the brains of aged rats following feeding control diets or diets enriched with GF formulas. **(C)** Quantified levels of iba-1 in the brains of aged rats following feeding control diets or diets enriched with GF formulas. N = 20 per group. P<0.001.

As seen in Fig. 7B-C, both microglial (iba-1) and glial fibrillary acidic protein (GFAP) were increased in the GF supplemented groups compared to the control group. In Figure 7A, a representative western blot for iba-1 immunoreactivity is shown. At 12 week supplementation with GF diets, all the aged rats exhibited lower iba-1 and GFAP expression compared to that seen in aged control rats as evidenced by semi-quantitative densitometry measurements. In the brains from GF supplemented animals, iba-1 expression was normalized to 100% while the GF treated animals decreased to around 23 +/− 8% post treatment (Fig. 7A and B). In addition, GFAP expression was 100% while the GF treated animals decreased to 14 ± 7% at 12 weeks post-supplementation, representing significantly reduced astrogliosis compared to GFAP in aged rats on a control diet (Fig. 7C).

### Nrf2 expression in aged rats following nutraceutical treatment

**Figure 8:**
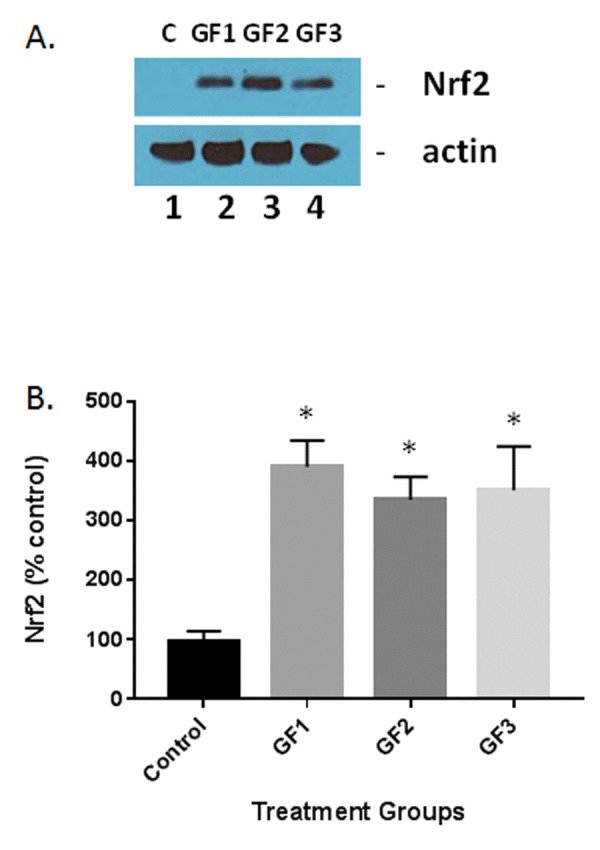
Measurement of Nrf2 expression in the aged brain and brains of animals fed GF diets (A) Representative western blot of Nrf2 levels in the brains of aged rats subjected to control or GF diets. C, control; GF1, GF2, GF3, GF diets. Actin is used as a control for loading. (B) Quantified levels of Nrf2 in the brains of aged rats following feeding control diets or diets enriched with GF formulas. N = 20 per group. P<0.001.

A number of studies have shown that polyphenolic chemicals are capable of activating the adaptive stress response pathways in various cells [26]. Of these responses, Nrf2 has been shown to play a critical role in the stress pathway by attenuation of the inflammatory process [27]. Western blot analysis of the Nrf2 in the brain of aged animals in the presence of GF diets or control diet was examined. As seen in Figure 8, the GF diets significantly increased Nrf2 expression in the brain. Quantification of the levels of Nrf2 showed that the GF diets increased Nrf2 by 300-400% over the 16 weeks of treatment (Fig. 8B). As demonstrated in Figure 7, both iba-1 and GFAP as well as other inflammatory cytokines were decreased in the presence of the GF diets suggesting an impact for Nrf2 on stress in aging. Decreases in MDA and DCF fluorescence and an increase in SOD are also indicators of Nrf2 activity. These data support the assumption that the activity is derived from the activation of antioxidant response element pathways from most cell types in the brain.

## DISCUSSION

The current study has shown that a diet enriched with GrandFusion^®^ can reduce inflammation and oxidative stress, neural stem cell proliferation and improved cognitive function in the aged rat brain. In addition, the treatment showed a significant increase in stem cell survival and activation of BDNF and Nrf2 to stimulate the expression of antioxidant genes. No significant differences were detected between male and female animals in the study. The overall premise is that aging exacerbates inflammation and that impacts the neuronal cells in the brain to alter their function and to stimulate gliosis that results in cognitive impairment.

The processes underlying age-related cognitive deficits persist to be fully depicted, it is clear that inflammation and oxidative stress play a significant role [28,29]. During the aging process, the CNS endogenous anti-inflammatory defenses deteriorate, causing the brain to be vulnerable to inflammatory insults [30-32]. The brain has limited regenerative capacity and various insults lead to aggregate cellular impairment that compromises normal neuronal and glial function. Mitochondrial dysfunction, lipid peroxidation, protein oxidation and altered autophagy are only some of the mechanisms responsible for intracellular damaged protein/lipid complexes, protein oligomers/aggregates, and cellular organelles [33]. These processes are well-regulated whose disruption leads to neuronal dystrophy and death, and are potential mechanisms of the aging process and the development of neurodegenerative diseases [34,35]. Neuroinflammation has been identified as a cause of neuronal dysregulation in aged rats [36]. Therefore, a potentially valuable approach to combat age-related deficits in behavior is to alter inflammation.

In the realm of aging, changes such as increased oxidative stress, inflammation characterized by reactive astrocytes and activated microglia and altered neurogenesis characterized by atypical migration of newly born neurons have been determined [37-41]. Memory and mood impairments in the aging are been attributed to the decline in neurogenesis and loss of neurons in the hippocampus [42,43]. Anti-aging therapies (AAT) can slow the aging process in most instances but cannot satisfactorily suppress age-induced detrimental changes described above [44-46]. Since these changes contribute to the aging process, anti-aging therapies have mostly failed to prevent the evolution of aging into chronic aging. Therefore, the best approach for aging should be capable of limiting neuronal loss, oxidative stress, inflammation and abnormal neurogenesis [47-50]. In this context, complexes and drugs having neuroprotective and/or anti-aging properties are excellent for preventing age-induced chronic neuronal dysfunction typified by aging, cognitive and mood impairments.

Other potential mechanisms for the impact of aging on cognition and memory may involve activation of cell signaling pathways such as p38 mitogen-activated protein kinase (MAPK), c-jun NH_2_-terminal kinase (JNK), caspase 1, and NFkB [51-56]. These kinases and factors occur within, and between, multiple signaling cascades that play a role in inflammation-associated cognitive deficits [57]. Interestingly, these pathways also interconnect with other pathways that are used by the neurotrophic factors, specifically BDNF. BDNF plays a critical role in the survival and development of certain populations of neurons [58]. In addition, BDNF can be neuroprotective, through the mitigation of damaging effects of a number of insults [59]. Finally, BDNF can play an important role in synaptic plasticity and cognition similar to inflammatory mechanisms associated with consolidation of hippocampus-dependent memory [60-62].

Studies from our group and others have implicated protection from aging and neurological disorders by increasing the dietary intake of fruits and vegetables [21-25,63]. Diets enriched in polyphenolics, which are high in antioxidant and anti-inflammatory compounds, such as blueberries, strawberries and spinach, can slow or even prevent the changes in neurochemical and behavioral parameters that characterize the aged brain [23,64-70]. We have demonstrated in several studies that diets supplemented with GrandFusion^®^, a mixture of fruits and vegetables, highly enriched in vitamins were able to limit the extent of cerebral ischemia injury and reverse several parameters of stroke, such as inflammation and oxidative stress and behavioral changes [21]. Finally, in addition to having anti-oxidant and anti-inflammatory properties, diets containing vegetables and fruits may have direct and indirect effects on cell signaling to augment neuronal communication, the ability to stimulate neurogenesis, enhancement of neurotrophic factor expression and the diminution of NF-κB [71-78]. Neurogenesis occurs in a number of locations including the dentate gyrus [68,69,72,77]. These cells migrate to areas of injury due to trauma or aging that helps to enhance neuronal function embracing cognition and motor function [72,77]. Therefore, neurogenesis in the dentate gyrus may lead to improved outcomes.

In summary, over the years, research has shown that changes in inflammation, oxidative stress and neurogenesis are involved in the aging process and therapeutics that alters this process can improve outcomes. The process of aging results in the elaboration of inflammatory cytokines and chemokines both present in the circulation as well as at the tissue level, which express their influence on neural stem cell population limiting their potential for regeneration and repair of the aging nervous system [23]. In addition, the presence of the inflammatory cytokines can affect neuronal function, induce microglial activation and astrocyte stimulation which can enhance the aging process [21]. The presence of GrandFusion® enriched in phyotchemicals and anti-inflammatory molecules help to attenuate inflammation and enhance neural stem cell survival and proliferation. This process helps to improve organ function, enrich the aging process and enhance cognitive ability in the animals.

## MATERIALS AND METHODS

### Animals and diets

Male and female Fischer 344 rats were purchased from Charles River Labs at 17 months of age. Rats were used at eighteen months of age. The rats were maintained on a 12-hour light/dark schedule in standard cages with ad libitum food and water. After 4 weeks of acclimation, rats were weight-matched and randomly assigned to diet groups (n = 15/group for both male and female groups). Food intake was recorded weekly and body weights were recorded every other week. Rats were monitored for signs of injury or disease in accordance with the Medical University of South Carolina (MUSC) and Ralph H. Johnson VA Institutional Animal Care and Use Committee. Dr. Kindy moved to the University of South Florida after the studies were completed. Animals were fed for 4 months (old animals) on a normal diet or a normal diet with ~2% supplementation of the different materials NF-216 (GrandFusion – Fruit and Veggie #1 Blend, GF1), NF-316 (GrandFusion – Fruit #2 Blend, GF2), NF-416 (GrandFusion – Vegetable #3 Blend, GF3) as described previously (21). The amount of supplementation was based on the recommended intake of the diet for human consumption. The supplementation was pulverized and incorporated into the pelleted diet. GrandFusion^®^ supplements were prepared by NutriFusion, LLC (www.nutrifusion.com). Average food intake was 0.020 ± 0.1 kg/kg body weight/day; and the average consumption of diets was 0.0004 ± 0.00005 kg/kg body weight/day. A food intake assessment (over a 96-h period) was made during the 8^th^ week of the experimental feeding. During the course of the study, no rats had to be removed from the study due to animal issues. All studies were approved that the Institutional Animal Care and Use Committee at the Medical University of South Carolina and the Veterans Affairs Medical Center.

### Histological examination

For histological examination, the animals were anesthetized with an intraperitoneal injection of sodium pentobarbital (50 mg/kg) at the indicated times. The brains were transcardially perfused with 4°C, 10% phosphate buffered saline (PBS). The brains were removed and chilled for 15 min at −20°C before being placed in a Rodent Brain Matrix. Coronal sections (2-mm thickness) were prepared and placed in OCT medium and frozen [25]. 30 μm sections were prepared for immunohistochemical analysis.

### ELISA analysis

For quantitative analysis of cytokines, an enzyme-linked immunosorbent assay (ELISA) was used to measure the levels of tumor necrosis factor-*α*, interleukin-1*β*, or interleukin-6 in brain tissue [79,80]. For BDNF assays, we used the ELISA kit provided by Abnova. Cytokines were extracted from rat brains as follows: frozen hemibrains were placed in Tissue Homogenization Buffer containing Protease Inhibitor Cocktail (PIC, Sigma, St Louis, MO, USA) 1:1,000 dilution immediately before use, and homogenized using polytron. Tissue sample suspensions were distributed in aliquots and snap frozen in liquid nitrogen for later measurements. ELISA kits were then used, according to manufacturer directions (Abnova, Taipei City, Taiwan).

### Behavioral assessment

The battery of motor tests and the cognitive test selected for this study have been previously shown by our group to be age-sensitive [21]. For most of the measures, the decline in performance is observed as early as 12 to 15 months of age. Previous work has shown that an N of 15 per group will give us sufficient power to find statistically significant (*P* < .05) differences on tests of behavior. Power calculations show that, for the Morris water maze (MWM), a difference in means of 10 (e.g., 32 vs 22 seconds in latency to find the platform), when the SD is 10, would yield a power of 78%, while for the rod walking test, a difference in means of 4.5 (e.g., 8.1 vs 12.6 s in latency to fall), when the standard deviation is 4, would yield a power of 85%, using a sample size of 15.

### Motor testing

After 2 months of dietary intervention, rats underwent a battery of motor tests to assess for (1) Balance: rats were placed on a large plank (38 mm; counterbalanced) and a narrow rod (26 mm) horizontally suspended 23 cm above a thick foam-core pad and latency to fall was recorded (max 60 seconds); and (2) Fine motor coordination and stamina: rats were placed on an accelerating rotarod (San Diego Instruments, San Diego, CA) consisting of a slowly accelerating (+2 rpm/30 s; 20 rpm max) rotating dowel (7 cm diameter) and latency to fall was recorded (max 300 seconds).

### Cognitive testing

To assess spatial learning and memory, rats completed a working memory version of the MWM (wMWM) at weeks 9–10 of dietary intervention [81,82]. The test was given daily for 4 consecutive days, 2 sessions per day and 2 trials each session: a reference memory or acquisition trial (trial 1) and a working memory or retrieval trial (trial 2). During testing, rats were placed in a large water-filled pool (134 cm diameter) and allowed 120 s to escape onto a platform hidden 2 cm below the water surface. If the rat failed to escape within this time, it was guided to the platform. Once the rat reached the platform, it was allowed to remain there for 15 s (trial 1). The rat was then returned to its home cage for 10 minutes (inter-trial interval). Trial 2 used the same platform location and start position as trial 1. The platform was moved to one of four locations, chosen to frustrate a number of non-place learning strategies that rats may adopt, at the beginning of each session. wMWM files were analyzed with image tracking software (San Diego Instruments, La Jolla, CA).

### Analysis of inflammation

Brain iba-1, GFAP, and actin (control) protein levels were determined at time of euthanasia. Animals were sham or treated with vehicle or supplemental diets. Relative levels of iba-1, GFAP, and actin in the supernatant fraction from the brain extract were determined by western blot analysis (polyclonal antibodies: iba-1 (sc-28530); GFAP, (sc-6170); β-actin (sc-130657); Santa Cruz Biotechnology, Santa Cruz, CA), as described previously [21]. For Nrf2 analysis, we used antibodies provided by Abcam (ab137550). Relative intensities of western blot bands were assessed by densitometry in triplicate for each sample. Densitometric analysis was done using IQTL software (GE Life Sciences, Piscataway, NJ). For protein studies, the entire lesional area was harvested for western blot analysis. In control or sham animals, a similar region was harvested.

### Thiobarbituric acid reactive substances

MDA (malondialdehyde) was evaluated by thiobarbituric acid reactive substances (TBARS) test [83]. Aliquots of samples were incubated with 10% trichloroacetic acid and 0.67% thiobarbituric acid. The mixture was heated (30 minutes) on a boiling water bath. Afterwards, n-butanol was added and the mixture was centrifuged (1,000 x g for 10 minutes). The organic phase was collected to measure fluorescence at excitation and emission wavelengths of 515 and 553 nm, respectively. 1,1,3,3-tetramethoxypropane, which is converted to MDA (malondialdehyde), was used as standard. Results are expressed as pmol MDA/mg protein and reported as percentage of control.

### Free radical levels

To assess the free radicals content, we used 20-70-dichlorofluorescein diacetate (DCFH-DA) as a probe [84]. A sample aliquot was incubated with DCFH-DA (100 mM) at 37°C for 30 minutes; the reaction was terminated by chilling the reaction mixture in ice. The formation of the oxidized fluorescent derivative (DCF) was monitored at excitation and emission wavelengths of 488 and 525 nm, respectively, using a fluorescence spectrophotometer (Hitachi F-2000, Hitachi High-Tech, Northridge, CA, USA). The free radicals content was quantified using a DCF standard curve and the results were expressed as pmol of DCF formed/mg protein. All procedures were performed in the dark and blanks containing DCFH-DA (no homogenate) were processed for measurement of autofluorescence.

### SOD assay

For the analysis of superoxide dismutase (SOD) activity, tissues were homogenized on ice in cold 25 mM HEPES buffer (pH 7.4) containing 250 mM sucrose and 1 mM EDTA and then centrifuged at 1500× g for 5 min at 4 °C. The activity of SOD was measured in supernatants using a superoxide dismutase assay kit following the manufacturer's instructions and expressed as units per mg of protein (Cayman Chemicals, Ann Arbor, MI). One unit is defined as the amount of enzyme needed to produce 50% dismutation of the superoxide radical.

### Protein determination

Protein was measured by the Coomassie blue method using bovine serum albumin as standard.

### Statistical analysis

The results were expressed as the mean±standard deviation (SD). The statistical significance of the results in the brain by ELISA, behavioral studies, physiological and histological data were analyzed using a T-test or one-way analysis of variance (ANOVA) followed by Fisher's post hoc test. Repeated-measures ANOVA were computed on the monitoring data and the significance of the difference among groups were evaluated by Fisher's post hoc test.

## Abbreviations

GF: GrandFusion
OS: oxidative stress
ROS: reactive oxygen species
MDA: malondialdehyde
TBARS: thiobarbituric acid reactive species
SOD: superoxide dismutase
BDNF: brain derived neurotrophic factor
GFAP: glial fibrillary acidic protein
AAT: anti-aging therapies.
